# Clinical outcome of Mantle Cell Lymphoma patients with high-risk disease (high-risk MIPI-c or high p53 expression)

**DOI:** 10.1038/s41375-023-01977-y

**Published:** 2023-07-26

**Authors:** Gabriel Scheubeck, Linmiao Jiang, Olivier Hermine, Hanneke C. Kluin-Nelemans, Christian Schmidt, Michael Unterhalt, Andreas Rosenwald, Wolfram Klapper, Andrea Evangelista, Marco Ladetto, Mats Jerkeman, Simone Ferrero, Martin Dreyling, Eva Hoster

**Affiliations:** 1 Department of Medicine III, LMU University Hospital, Munich, Germany; 2 Institute for Medical Information Processing, Biometry, and Epidemiology, LMU Munich, Munich, Germany; 3 University Hospital, Paris, France; 4 Department of Hematology, University Medical Center Groningen, University of Groningen, Groningen, Netherlands; 5 Institute of Pathology, University of Wuerzburg, Wuerzburg, Germany; 6 Department of Pathology, Hematopathology Section, University Hospital Schleswig-Holstein, Campus Kiel, Kiel, Germany; 7 Unit of Clinical Epidemiology, Azienda Ospedaliera Universitaria Città della Salute e della Scienza and CPO Piemonte, Turin, Italy; 8 Hematology, Azienda Ospedaliera SS Antonio e Biagio e Cesare Arrigo, Alessandria, Italy; 9 Skane University Hospital, Lund, Sweden; 10 Division of Hematology, Department of Molecular Biotechnologies and Health Sciences University of Torino/AOU “Città della Salute e della Scienza di Torino”, Turin, Italy

**Keywords:** B-cell lymphoma, Biostatistics

## Abstract

Currently, treatment allocation of patients with Mantle Cell Lymphoma (MCL) is mainly based on age and medical fitness. The combined MCL International Prognostic Index (MIPI-c) allows to predict prognosis using clinical factors (MIPI) and the Ki-67 index. However, high p53 expression as surrogate for *TP53* alterations has demonstrated to be an independent predictor for poor outcome. We aimed to define a clear high-risk group based on the combination of MIPI, Ki-67 and p53 expression/*TP53* alteration. A total of 684 patients from the prospective European MCL-Younger and MCL-Elderly trials were evaluable. The classification of high-risk disease (HRD) as high-risk MIPI-c or p53 expression >50% versus low-risk disease (LRD) as low, low-intermediate or high-intermediate MIPI-c and p53 expression ≤50% allowed to characterize two distinct groups with highly divergent outcome. Patients with HRD had significantly shorter median failure-free survival (FFS) (1.1 vs. 5.6 years, *p* < 0.0001) and overall survival (OS) (2.2 vs. 13.2 years, *p* < 0.0001) compared to those with LRD. These major differences were confirmed in two validation cohorts from the Italian MCL0208 and the Nordic-MCL4 trials. The results suggest that this subset of HRD patients is not sufficiently managed with the current standard treatment and is asking for novel treatment strategies.

## Introduction

Mantle cell lymphoma (MCL) is a rare and commonly aggressive subtype of B-cell lymphoma characterized by the translocation t(11;14) with consecutive cyclin D1 overexpression. The clinical course is heterogeneous and marked by recurring relapses. Novel therapeutic strategies such as the addition of high-dose cytarabine to the induction treatment prior to autologous stem cell transplantation (aSCT), Rituximab (R) maintenance and the approval of Bruton’s tyrosine kinase inhibitors (BTKi) substantially improved the survival of patients [1–3]. Currently, patients are allocated for treatment mainly considering age, stage and performance status.

The Mantle Cell Lymphoma International Prognostic Index (MIPI) allows for discriminating prognostic risk groups based on age, performance status, lactate dehydrogenase (LDH) and leukocyte count [4], the combined MIPI-c additionally incorporates the Ki-67 index [5]. In prospective trials, the biological risk factors Ki-67, blastoid or pleomorphic cytology [5], and *TP53* alterations [6–8] were associated with inferior outcome independent of MIPI. The prognostic relevance of MCL cytology is closely correlated to the Ki-67 index, which is generally increased in blastoid MCL [5, 9, 10]. P53 expression is a widely applicable diagnostic method serving as surrogate marker for *TP53* alterations [11, 12]. In univariate analyses *TP53* alterations, such as mutations and deletions, were both validated as negative predictor for outcome in the Nordic MCL2, MCL3, and MCL4 as well as in the European MCL Younger and Elderly trial cohort [7, 8, 13]. Thus, even more intensive regimens including high-dose cytarabine and aPBSCT fail to overcome the dismal prognosis of *TP53* alterations [7, 8].

New concepts are urgently needed to define a more refined high-risk population and identify effective treatment strategies for these patients. In this study, we aimed to define a combination of MIPI, Ki-67 and p53 expression/*TP53* alterations that reliably identifies a high-risk group.

## Methods

### Patients

A total of 1183 MCL patients with confirmed MCL and Ann Arbor stage II to IV were registered in the MCL Younger [14] (NCT00209222) and MCL-Elderly trial [15] (NCT00209209) of the European MCL Network from 2004–2010 serving as training cohort. Patients without available Ki-67 and p53 data were excluded from this analysis. All patients gave written informed consent to participation in the trials.

Two independent series of 300 patients from the MCL0208 trial [16] and 51 patients from the Nordic-MCL4 trial [17] were included in this study as validation cohort. The MCL0208 cohort considered TP53 mutation or del(17p) and the Nordic-MCL4 cohort only TP53 mutation status instead of p53 expression data.

### Immunohistochemistry

Immunohistochemistry was performed centrally on either tissue microarrays or whole tissue sections. P53 was stained with a mouse monoclonal antibody (Leica/NovoCastra clone DO7) and scored as negative (0%), low (1–10%), intermediate (10–50%) or high (>50%) by one observer based on visual assessment as described [6]. Ki-67 index was centrally assessed in accordance with established guidelines [18].

### Pathology

Formalin-fixed paraffin-embedded (FFPE) diagnostic biopsy material was classified as classical or pleomorphic/blastoid variant by cytomorphological features confirmed by the central pathology review of a pathology reference center (European MCL Pathology Panel).

### Risk variables

Biological risk variables Ki-67 and p53 expression along with the clinical prognostic tool MIPI were investigated in various combinations. MIPI score is the weighted sum of MIPI prognostic factors weighted by the regression coefficients from the defining Cox regression model [4]. The equally weighted combination of MIPI with the dichotomized Ki-67 index at the validated 30% cutoff defines the MIPI-c [5].

It was previously reported that none of the patients in the MCL Younger and Elderly cohort with complete absence of p53 expression did display *TP53* deletions [6, 8]. For this reason, we focused on the cutoff for p53 expression at 50% serving as biomarker indicating a *TP53* mutation with altered functions [11, 19].

Combinations tested to define high-risk disease (HRD) were p53 expression >50% or Ki-67 ≥ 30% (definition 1), p53 expression >50% or high, high-intermediate MIPI-c (definition 2), or p53 expression >50% or high MIPI-c (definition 3). Accordingly, low-risk disease (LRD) was defined by p53 expression ≤50% and Ki-67 < 30% (definition 1), p53 expression ≤50% and low, low-intermediate MIPI-c (definition 2) or p53 expression ≤50% and low, low-intermediate, high-intermediate MIPI-c (definition 3).

For the validation cohort, presence of TP53 mutation and del(17p) (only MCL0208 cohort) defined HRD instead of high p53 expression.

### Statistical methods

Cases with missing data for both Ki-67 and p53 expression or TP53 mutation/deletion, respectively, were excluded from the analysis. If one high-risk feature applies, missing data for the other variable is allowed. For the classification of LRD all variables must be available. The percentages of HRD in the study population were estimated based on complete cases, where patients with missing data in either Ki-67 or p53 expression were excluded in order to minimize bias. The number of complete cases is significantly smaller but is more reliable to estimate the true proportion of MCL patients with HRD. Analyses for the outcomes were performed on all the classifiable patients with available outcome data. In addition, we performed sensitivity analyses with complete cases.

We estimated and compared failure-free survival (FFS, defined as time from treatment start to stable disease, progression, or death from any cause, whichever occurred first) and overall survival (OS, defined as time from study registration to death from any cause) stratified by Ki-67 index (</≥30%), cytology (blastoid versus non blastoid), p53 expression (≤/>50%), the combination of Ki-67 and p53 and MIPI-c and p53 using Kaplan-Meier-plots, logrank tests, and Cox regression. Five-year FFS and OS probabilities were reported along with median FFS and OS times. Quantification of follow-up was done by the reversed Kaplan-Meier method. Status of the clinical data was that of July 08, 2021, the latest available time point of medically reviewed data from MCL Younger and MCL-Elderly trials.

## Results

Six hundred eighty-four patients (MCL Younger *n* = 390, MCL Elderly *n* = 294) out of 1183 registered study patients with MCL from the training cohort had evaluable data either for Ki-67 or p53 (Supplemental Fig. 1). Among these, low-risk MIPI was more frequent (43% vs. 27%), whereas high-risk MIPI was significantly less frequent (24% vs. 44%, *p* < 0.0001) compared to those without evaluable data (Table 1). Accordingly, median FFS and OS was superior in the subgroup of patients with available data (4.4 vs. 3.2 years, *p* = 0.0066 and 9.6 vs. 6.6 years, *p* = 0.0013) (Supplemental Fig. 2). This difference is mainly explained by an overrepresentation of patients with available pathology data from the MCL Younger trial (57% vs 45%) who had a better overall outcome. Of note, there was no difference in outcome of patients with a high MIPI comparing those with available data for Ki-67/p53 vs. unavailable data for Ki-67 and p53.

**Table 1:** Characteristics of patients with available vs. unavailable data for Ki-67 or p53.

Variable	Value	Ki-67/p53 available (*N* = 684)		Ki-67 and p53 unavailable (*N* = 499)		*P* value
Study	MCL Younger (*n*, %)	390	57%	223	45%	<0.0001
Induction	CHOP + ACD20^a^ (*n*, %)	150 (*n* = 671)	22%	138 (*n* = 477)	29%	<0.0001
	R-FC (*n*, %)	143 (*n* = 671)	21%	130 (*n* = 477)	27%	
	R-CHOP/R-CHOP (*n*, %)	163 (*n* = 671)	24%	73 (*n* = 477)	15%	
	R-CHOP/R-DHAP (*n*, %)	214 (*n* = 671)	32%	135 (*n* = 477)	28%	
Age (years)	Median, Min-Max	62	30–85	66	36–88	<0.0001
Sex	Male (*n*, %)	519	76%	372	75%	0.63
Stage	I (*n*, %)	2	0%	2	0%	0.13
	II (*n*, %)	37	5%	21	4%	
	III (*n*, %)	89	13%	46	9%	
	IV (*n*, %)	556	81%	430	86%	
Bone marrow	Involved (*n*, %)	512	75%	390	78%	0.19
B-symptoms	Present (*n*, %)	245	36%	192	38%	0.36
ECOG	2–4 (*n*, %)	39	6%	27	5%	0.90
LDH (ULN)	Median, Min–Max	0.92	0.29–12.22	0.94	0.29–11.27	0.10
WBC (G/L)	Median, Min–Max	7.28	1.04–1105	8.5	1.05–805	<0.0001
Ki-67	Median, Min–Max	20 (*n* = 612)	0–97	–	–	–
Ki-67	≥30%	180 (*n* = 612)	29%	–	–	–
Cytology	blastoid	56 (*n* = 525)	11%	9 (*n* = 126)	7%	0.32
MIPI score	Median, Min–Max	5.8	4.21–8.68	6.1	4.07–8.84	<0.0001
MIPI	Low (*n*, %)	293	43%	134	27%	<0.0001
	Intermediate (*n*, %)	225	33%	146	29%	
	High (*n*, %)	166	24%	219	44%	
MIPI-c	Low (*n*, %)	201 (*n* = 612)	33%	–	–	–
	Low intermediate (*n*, %)	205 (*n* = 612)	33%	–	–	
	High intermediate (*n*, %)	143 (*n* = 612)	23%	–	–	
	High (*n*, %)	63 (*n* = 612)	10%	–	–	
P53 expression	0% (*n*, %)	42 (*n* = 348)	12%	–	–	–
	1% - 10% (*n*, %)	157 (*n* = 348)	45%	–	–	
	10% - 50% (*n*, %)	95 (*n* = 348)	27%	–	–	
	> 50% (*n*, %)	54 (*n* = 348)	16%	–	–	
Risk group	High-risk (*n*, %)	109 (*n* = 325)	34%	–	–	–

^a^ACD20: anti CD20 antibody.

Apart from MIPI parameters, patient characteristics were equally distributed among the two groups (Table 1). High MIPI-c was seen in 63 of 612 cases (10%) and high p53 expression in 54 of 348 (16%) cases. High p53 expression was associated with inferior median FFS (1.5 vs. 4.6 years; *p* < 0.0001) and OS (2.8 vs. 10.7 years, *p* < 0.0001) compared to p53 expression ≤50% (data not shown). Blastoid cytology was no negative predictor in patients with low Ki-67 (Supplementary Fig. 3).

Using Ki-67 ≥ 30% or high p53 expression to define HRD (definition 1) resulted in a relatively large high-risk group with 37% of complete cases. Median FFS and OS was 1.8 vs. 6.0 years (HR 2.01, *p* < 0.0001) and 4.0 vs. 14.4 years (HR 2.57, *p* < 0.0001) compared to LRD (Supplemental Fig. 4). Considering also clinical factors, we tested the impact of high, high-intermediate MIPI-c or high p53 expression (definition 2) on outcome. This high-risk definition includes 41% of complete cases and revealed similar results (median FFS: 1.7 vs. 6.0 years, HR 2.40, *p* < 0.0001; median OS: 3.6 vs. 15.4 years, HR 3.24, *p* < 0.0001) (Supplemental Fig. 5).

**Fig. 1:**
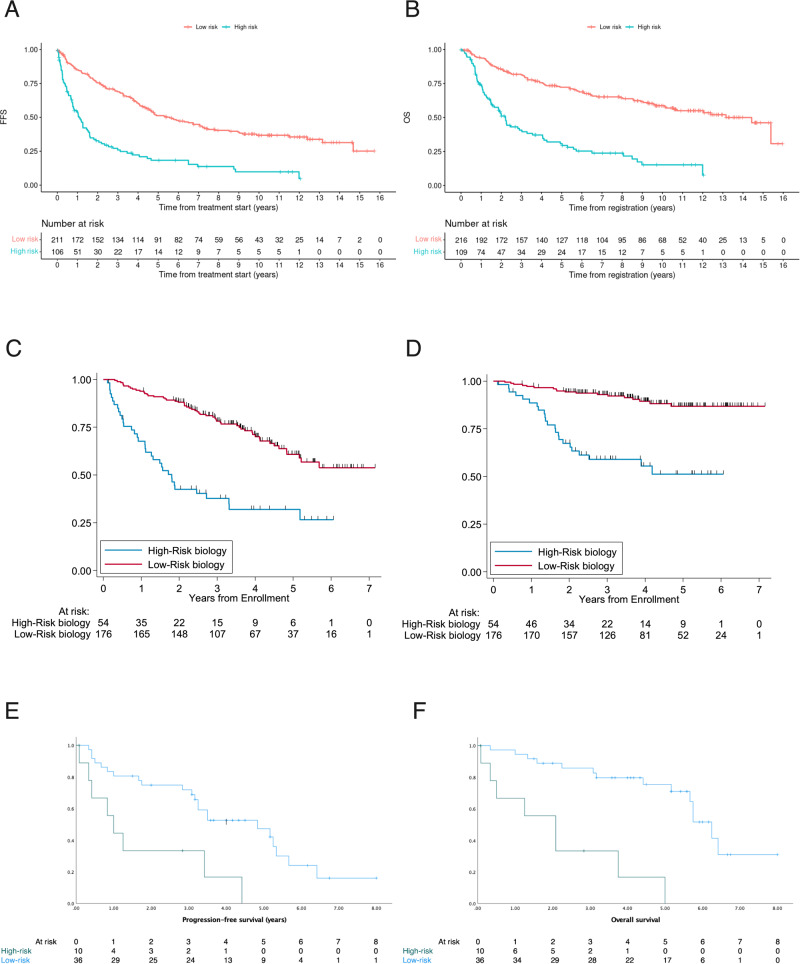
Failure-free and overall survival of HRD compared to LRD MCL patients. Prognostic impact of high MIPI-c or high p53 expression/*TP53* mutation (definition 3) in the training (**A, B**) and the validation cohorts (**C**–**F**). Kaplan–Meier estimates of FFS (**A**) and OS (**B**) of patients with high MIPI-c or p53 expression >50% (high-risk disease) compared to low, low-intermediate or high-intermediate MIPI-c and p53 expression ≤50% (low-risk disease) from the MCL Younger and Elderly cohort. The number at risk is based on all classifiable patients. Estimates of PFS and OS in the MCL0208 (**C**, **D**) and the MCL4 (**E**, **F**) validation cohorts of patients with high MIPI-c or *TP53* mutation and del(17p) (only MCL0208 cohort) (high-risk disease) compared to patients with low, low-intermediate or high-intermediate MIPI-c and no *TP53* mutation and del(17p) (low-risk disease).

**Fig. 2:**
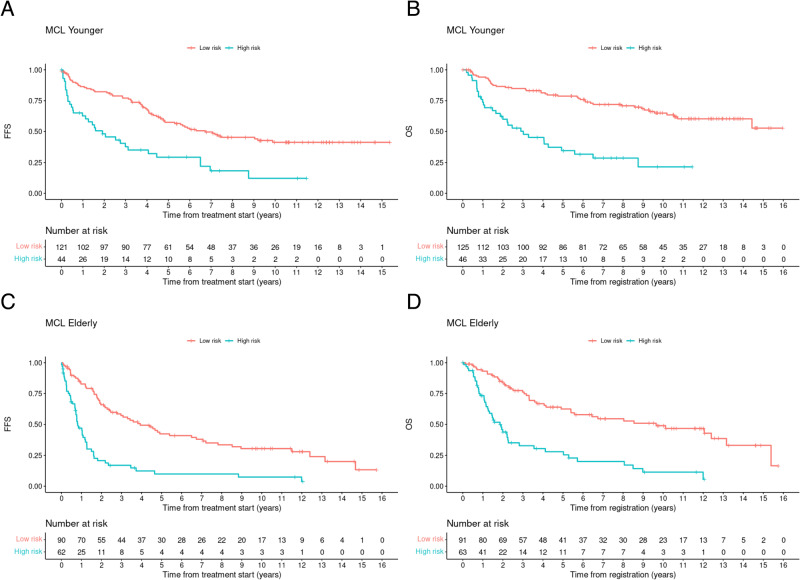
Prognostic impact of high MIPI-c or high p53 expression on outcome stratified by trial groups. Kaplan–Meier estimates of FFS (**A**, **C**) and OS (**B**, **D**) among patients treated in the MCL Younger (**A**, **B**) and MCL Elderly (**C**, **D**) trial stratified by the presence of high MIPI-c or p53 expression >50% (high-risk disease) compared to low, low-intermediate or high-intermediate MIPI-c and p53 expression ≤50% (low-risk disease). The number at risk is based on all classifiable patients.

The combination of high MIPI-c or high p53 expression (definition 3) defined the smallest group of high-risk patients and had the highest discriminatory power between HRD and LRD in terms of the hazard ratios for FFS and OS, why we chose that definition for further analyses. Based on the selection process of definition 3, 22% complete cases could be assigned to the HRD group (*n* = 60) and 78% (*n* = 216) had confirmed LRD. After a median follow-up of 9.6 (FFS) and 9.4 (OS) years the median FFS (1.1 years vs. 5.6 years; HR 2.97, *p* < 0.0001; Fig. 1A) and OS (2.2 vs. 13.2 years, HR 3.69, *p* < 0.0001; Fig. 1B) was significantly decreased in the high-risk compared with the low-risk group. 5-year and 10-year FFS probabilities were 18% vs. 51% and 10% vs. 37%; 5-year and 10-year OS probabilities were 31% vs. 72% and 15% vs. 59% for HRD vs. LRD, respectively. Sensitivity analyses in complete cases showed similar results to the analyses in classifiable cases. These aforementioned significant differences were observed consistently across both trial groups, regardless of whether patients received conventionally dosed immunochemo- and maintenance therapy in MCL Elderly (median FFS: 0.8 vs. 3.9 years, *p* < 0.0001; median OS: 1.9 vs 9.7 years, *p* < 0.0001) or received induction with intention to high-dose chemotherapy followed by aSCT in MCL Younger (median FFS: 1.9 vs. 6.7 years, *p* < 0.0001; median OS: 3.0 years vs. median not reached, *p* < 0.0001) (Fig. 2).

**Fig. 3:**
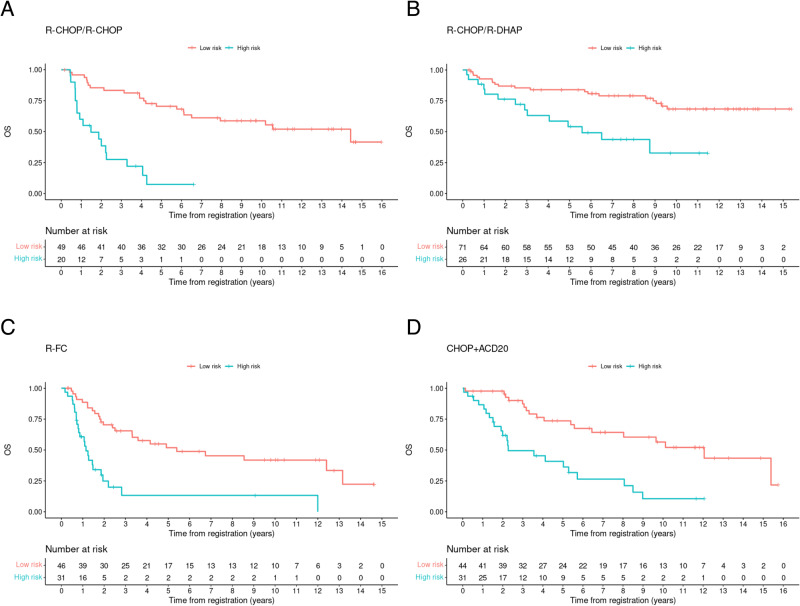
Prognostic impact of high MIPI-c or high p53 expression on overall survival stratified by treatment groups of the MCL Younger and the MCL Elderly trial. Kaplan–Meier estimates of OS among patients treated with R-CHOP induction and aPBSCT (**A**), alternating R-CHOP/R-DHAP induction and aPBSCT (**B**), R-FC and IFN maintenance (**C**) and R-CHOP + R maintenance (**D**) stratified by the presence of high MIPI-c or p53 expression >50% (high-risk disease) compared to low, low-intermediate or high-intermediate MIPI-c and p53 expression <50% (low-risk disease). The number at risk is based on all classifiable patients.

Subgroup analyses showed that R-CHOP compared to R-FC in MCL Elderly (median OS 1.1 vs. 2.3 years; HR 3.61 vs. 3.50) as well as R-CHOP/R-DHAP induction compared to R-CHOP induction in MCL Younger (median OS 1.5 vs. 5.6 years; HR 5. 94 vs. 3.06.) could partially mitigate the dismal prognosis of HRD. However, the superior treatment arms failed to fully compensate for the poor prognostic impact of HRD (Fig. 3).

The results were validated in independent series of 230 classifiable patients from the FIL-MCL0208 trial and 44 classifiable patients from the Nordic Lymphoma Group MCL4 trial. The MCL0208 trial enrolled patients aged 18–65 years undergoing cytarabine-containing induction and aSCT before randomization to 24 months of lenalidomide maintenance compared to observation [16]. The Nordic-MCL4 trial was carried out in patients >65 years not suitable for aSCT receiving the combination of lenalidomide and R-bendamustine [17]. HRD (definition 3) was present in 20% (MCL0208) and 23% (MCL4) of complete cases. Main patient features are shown in Supplemental Table 1 (MCL0208) and 2 (MCL4). Presence of HRD according to definition 3 conditioned significant inferior outcome in both, the MCL0208 cohort and the MCL4 cohort. Median Progression-free survival (PFS) was 1.8 years vs. 5.2 years (HR 3.52, *p* < 0.001; Fig. 1C) and 1.0 years vs. 4.8 years (HR 3.6, *p* = 0.002; Fig. 1E) for HRD vs. LRD in the MCL0208 and the MCL4 validation cohort. The adverse prognostic value also translated into OS with a hazard ratio for death of 5.5 (95% CI: 2.96–10.22, *p* < 0.001) (Fig. 1D) and 7.6 (95% CI: 2.8–20.8, *p* < 0.0001) (Fig. 1F) for HRD disease in the MCL0208 and the MCL4 series.

## Discussion

To the best of our knowledge, this is the first investigation exploring a novel combination of the biological and clinical risk factors Ki-67, p53 expression/*TP53* alterations and MIPI that is valid for young and elderly MCL patients. All patients were treated in prospective trials from the European Mantle Cell Lymphoma Network. The frequency of high MIPI-c and high p53 expression at 10% and 16% of the training cohort matches well with the Nordic MCL2 and MCL3 study cohort where the reported frequency of high MIPI-c and *TP53* mutations was 13% and 16%, respectively [7].

The p53 tumor suppressor gene (*TP53*) is a crucial regulator of the cell cycle, apoptosis, DNA repair and senescence [20]. Genetic aberrations of *TP53* such as point mutations and allelic deletions regularly emerge during tumorigenesis and result in a loss-of-function of the *TP53* gene [21]. Consequently, the p53 protein often accumulates which can be visualized using p53-specific antibodies [11]. DNA sequencing is considered the most reliable method to analyze the *TP53* mutation status. Immunohistochemistry has good correlation to *TP53* missense mutations, but still misses up 18% of these mutations [22]. On the other hand, immunohistochemistry misses truncating mutations (non-missense) that lead to the lack of expression of the protein. These mutations may be up to 10–25% of the TP53 mutations in MCL and may represent up to 11% of patients with low protein expression [22, 23]. Of note, some patients with high p53 expression were reported to have wild type *TP53*. Considering these limitations, in the era of precision medicine, we recommend the molecular study of *TP53* as already performed in clinical practice in chronic lymphocytic leukemia. However, p53 expression serving as surrogate for *TP53* mutation status is characterized by the wide and practical availability. Accordingly, we recommend to assess both TP53 mutation status and p53 expression by IHC for all MCL patients. If either the molecular study indicates deletion/mutation or p53 expression is >50%, the disease should be considered high-risk.

While high p53 expression was an independent risk factor for poor outcome, blastoid cytology had no predictive value in patients with low Ki-67 < 30%. These results confirm that adding cytology, which is known to be poorly standardized, to the definition of HRD does not relevantly improve the results. However, it must be noted, that of 341 patients with Ki-67 < 30% and available morphology, only 16 had blastoid morphology. This leads to limited power to detect an effect of blastoid cytology. We recommend maintaining the determination of MCL morphology in clinical practice.

Considering the two biological risk factors high Ki-67 or p53 expression (definition 1) identified patients with a 2.5-fold higher risk of death compared to those without these risk factors. Adding also clinical risk factors according to definition 2 revealed a group of high-risk patients with a 3.2-fold higher risk of death. Definition 3 distinguished most clearly between high and low-risk patients with a 3-fold higher risk of treatment failure and a 3.7-fold higher risk of death for HRD. The frequency of high-risk disease according to definition 3 was consistent in the training as well as in the validation cohort. The dismal outcome of this high-risk group with half of the patients failing treatment after one and dying after 2 years, is in line with the data reported for patients with mutated *TP53* in the MCL2 and MCL3 cohort [7]. The “MIPI-genetic” (“MIPI-g”) which was developed for younger MCL patients and adds *KMT2D* mutations and *TP53* disruptions to MIPI-c identifies a high-risk group with a 4-year PFS and OS probability of 11.5% and 44.9% [12].

Also in the superior treatment arms of the MCL-Elderly and Younger Trial, the presence of HRD reliably predicted poor survival.

The vast majority of the patients in the MCL-Elderly and Younger Trial received conventional chemotherapy after the first relapse [24, 25]. While allogeneic or autologous stem cell transplantation was carried out in some patients of the Younger trial, only a minority received targeted therapies such as a BTKi (1–3% in the MCL Younger, 7–10% in the MCL-Elderly trial) [24, 25]. Hence, the efficacy of novel therapies in HRD patients can hardly be inferred from these trials as the numbers are too small.

Of note, also the immunomodulatory drug lenalidomide trial did not overcome the adverse impact of *TP53* mutations in combination with R-bendamustine [13]. The general validity of the biology-based HRD model was confirmed in the FIL-MCL0208 and the Nordic-MCL4 trial with significant inferior PFS and higher risk for death in high-risk patients.

Interestingly, in relapsed or refractory MCL even the potent BTKi ibrutinib does not overcome the poor prognosis that is linked to *TP53* mutations and high MIPI-c [26–28]. A benefit of ibrutinib for progression-free survival in the first line treatment of elderly patients was recently reported in the SHINE study (ClinicalTrials.gov Identifier: NCT01776840) [29]. However, the addition of ibrutinib to rituximab and bendamustine did not show a clear benefit in patients with *TP53* mutations or a MIPI score indicating high-risk [29]. The benefit of ibrutinib in combination with chemotherapy in treatment-naive transplant eligible patients has been recently reported for the TRIANGLE study, but longer follow-up is required to evaluate whether this combination fully overcomes the biological risk factors [30]. Of note, neither Ki-67 ≥ 50%, *TP53* mutation, nor intermediate or high MIPI had any negative prognostic value for 6-months PFS in relapsed or refractory patients treated with the novel CD19 directed CAR-T-cell therapy KTE-X19 [31], suggesting that cellular immunotherapy might overcome the poor prognosis in high-risk patients.

As MCL patients with HRD defined by high MIPI-c or high p53 expression/*TP53* alteration had a dismal clinical course of the disease, we recommend to incorporate these factors in routine diagnostic practice as suggested by the WHO 5^th^ edition and the International Consensus Classification (ICC) to identify patients with need for novel therapeutic strategies [32, 33]. At present, HRD MCL patients should be treated with ibrutinib-containing induction based on the SHINE and TRIANGLE data [29, 30]. However, we think that this approach will not completely overcome the dismal prognosis of high MIPI-c or high p53/*TP53* mutation and clinical trials are needed that particularly address HRD patients.

In conclusion, the combination of the prognostic MIPI index with the biological risk factors *TP53* mutation and high Ki-67 expression reliably defines a subset of MCL patients with dismal prognosis. On the other hand, patients without these high-risk features achieve an excellent outcome with an overall survival over a decade with the current standard of care. Furthermore, these results will allow risk stratification in clinical trials, to hopefully develop innovative therapies especially for the high-risk MCL population which has the greatest medical need.

## Supplementary information

Supplemental tables and figures

## Data Availability

The datasets generated during and/or analyzed during the current study are available from the corresponding author on reasonable request.
