# Supplementary material for: Clinical outcome of Mantle Cell Lymphoma patients with high-risk disease (high-risk MIPI-c or high p53 expression)
Source: Leukemia. 2023 Jul 26;37(9):1887–94. doi: 10.1038/s41375-023-01977-y (PMC10457193; doi:10.1038/s41375-023-01977-y)
Supplement: Supplementary file 1 — Supplemental tables and figures [file 41375_2023_1977_MOESM1_ESM.pdf]

Supplement

Supplemental Tables

Supplemental Table 1: Demographic and clinical characteristics of MCL0208 patients at enrolment with available data for Ki-67 or TP53 mutation or del17(p)

Factor	Level	All	Low-risk disease	High-risk disease
**N**		230	176	54
**Age at enrolment, median (IQR)**		57 (52, 62)	57 (50, 62)	59 (54, 61)
**Sex**	Female	52 (22.6%)	39 (22.2%)	13 (24.1%)
	Male	178 (77.4%)	137 (77.8%)	41 (75.9%)
**Abnormal LDH**		74 (32.2%)	44 (25.0%)	30 (55.6%)
**ECOG**	0	181 (78.7%)	145 (82.4%)	36 (66.7%)
	1	41 (17.8%)	26 (14.8%)	15 (27.8%)
	2	8 (3.5%)	5 (2.8%)	3 (5.6%)
**Stage**	II	2 (0.9%)	2 (1.1%)	0 (0.0%)
	III	12 (5.2%)	8 (4.5%)	4 (7.4%)
	IV	216 (93.9%)	166 (94.3%)	50 (92.6%)
**Bulky disease (5 cm)**	NO	147 (63.9%)	120 (68.2%)	27 (50.0%)
	YES	83 (36.1%)	56 (31.8%)	27 (50.0%)
**Bone marrow involvement**		185 (80.4%)	139 (79.0%)	46 (85.2%)
**MIPI Risk Group**	Low	140 (60.9%)	122 (69.3%)	18 (33.3%)
	Intermediate	58 (25.2%)	47 (26.7%)	11 (20.4%)
	High	32 (13.9%)	7 (4.0%)	25 (46.3%)
**MIPI-c Risk Group**	Low	111 (48.3%)	99 (56.3%)	12 (22.2%)
	Low-Intermediate	64 (27.8%)	54 (30.7%)	10 (18.5%)
	Intermediate-High	30 (13.0%)	23 (13.1%)	7 (13.0%)
	High	23 (10.0%)	0 (0.0%)	23 (42.6%)
	Missing	2 (0.9%)	0 (0.0%)	2 (3.7%)
**Ki67 index >30%**		75 (32.9%)	39 (22.2%)	36 (69.2%)
**TP53mut or del(17p)**		38 (17.0%)	0 (0.0%)	38 (80.9%)

Supplemental Table 2: Demographic and clinical characteristics of MCL4 patients at enrolment with available data for Ki-67 or TP53 mutation

Factor	Level	All	Low-risk disease	High-risk disease
**N**		46	36	10
**Age at enrolment, median (IQR)**		72 (70, 77)	72 (70, 78)	72 (70, 76)
**Sex**	Female	12 (26.1%)	8 22.2%)	4 (40%)
	Male	34 (73.9%)	28 (77.8%)	6 (60%)
**Abnormal LDH**		18 (39.1%)	12 (33.3%)	6 (60%)
**ECOG**	0	21 (45.7%)	17 (47.2%)	4 (40%)
	1	22 (47.8%)	18 (50%)	4 (40%)
	2	3 (6.5%)	1 (2.8%)	2 (20%)

**Stage**	II	2 (4.3 %)	2 (5.6%)	0
	III	4 (8.7%)	4 (11.1%)	0
	IV	40 (87%)	30 (83.3%)	10 (100%)
**Bulky disease (5 cm)**	NO	N/A	N/A	N/A
	YES	N/A	N/A	N/A
**Bone marrow involvement**		40 (87%)	30 (83.3%)	10 (100%)
**MIPI Risk Group**	Low	5 (10.9%)	5 (13.9%)	0
	Intermediate	16 (34.8%)	15 (41.7%)	1 (10%)
	High	25 (54.3%)	16 (44.4%)	9 (90%)
**MIPI-c Risk Group**	Low	5 (10.9%)	5 (13.9%)	0
	Low-Intermediate	12 (26.1%)	12 (33.3%)	0
	Intermediate-High	23 (50%)	19 (52.8%)	4 (40%)
	High	6 (13%)	0 (0%)	6 (60%)
	Missing	-	-	-
**Ki67 index >30%**		10 (21.7%)	4 (11%)	6 (60%)
**TP53mut**		6 (11.8%)	0 (0%)	6 (60%)

Supplemental Figures

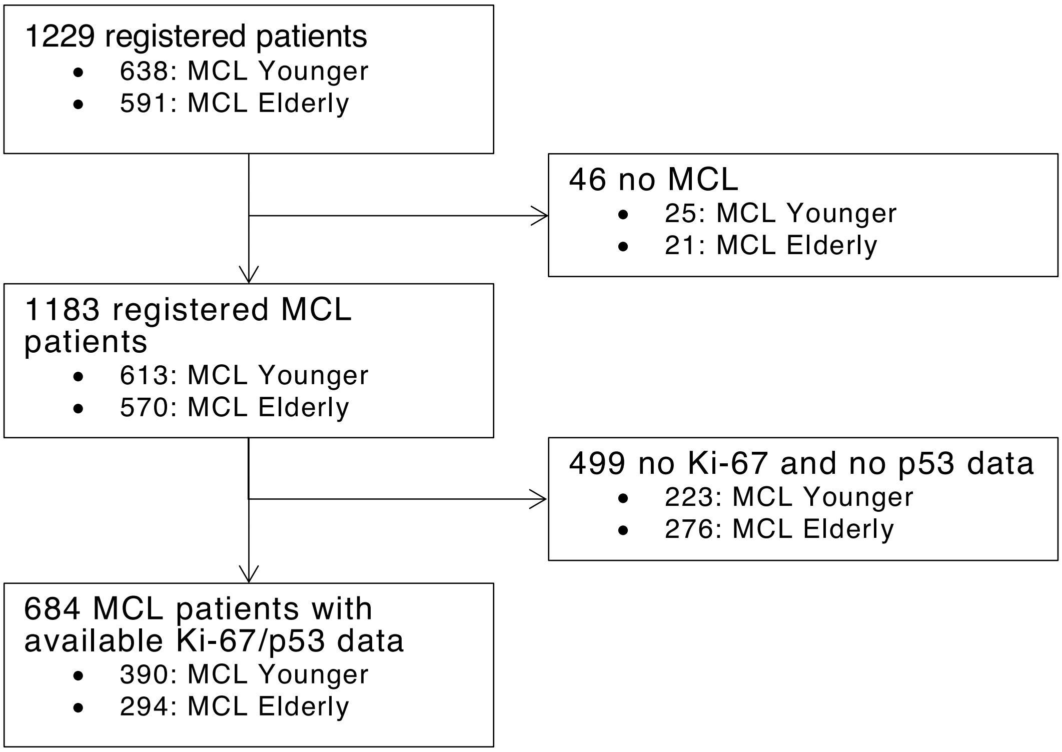

Supplemental Figure 1: Selection of patients for high-risk disease (HRD) assessment.

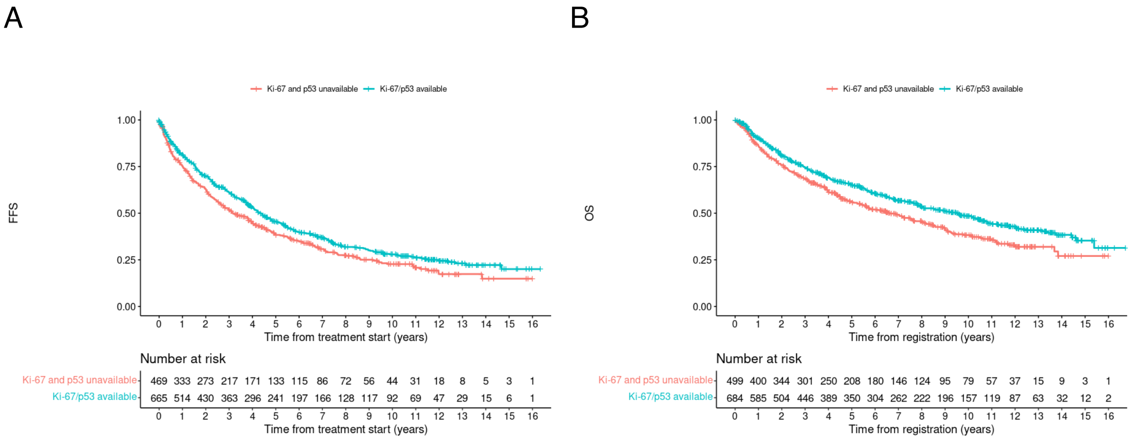

Supplemental Figure 2: **Outcome of patients with available data on Ki-67 or p53 expression compared to those without available data.** Kaplan-Meier estimates of FFS (A) and OS (B) stratified by patients with available data for Ki-67 or p53 compared to patients without available data for Ki-67 and p53.

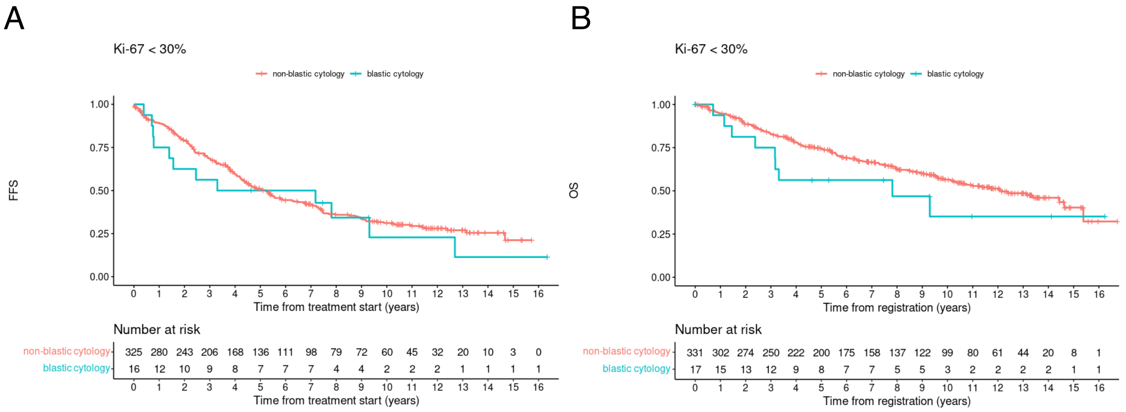

Supplemental Figure 3: Prognostic impact of MCL cytology in association with low Ki-67. Kaplan-Meier estimates of FFS (A) and OS (B) stratified by patients with blastoid or non-blastoid cytology adjusted for Ki-67 <30%.

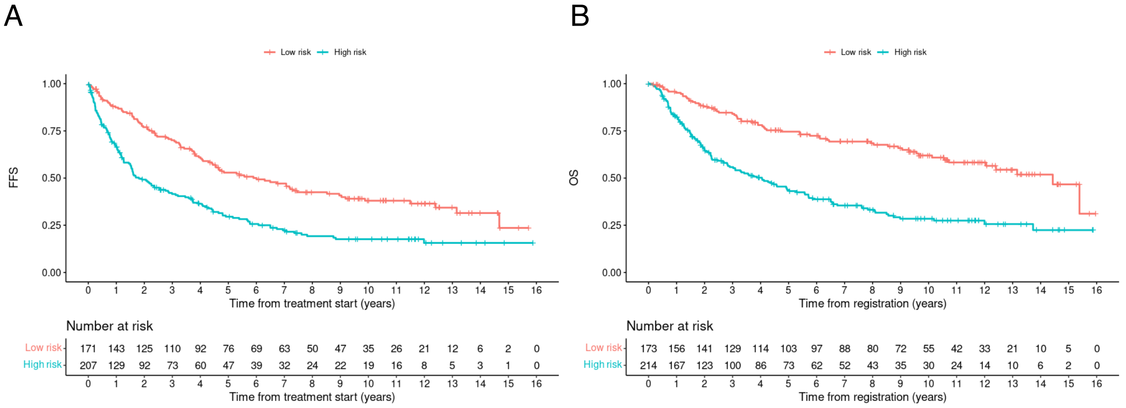

Supplemental Figure 4: Prognostic impact of high Ki-67 or high p53 expression on clinical outcome. Kaplan-Meier estimates of FFS (A) and OS (B) of patients with Ki-67 ≥30% or p53 expression >50% (high-risk disease) compared to Ki-67 <30% and p53 expression ≤50% (low-risk disease).

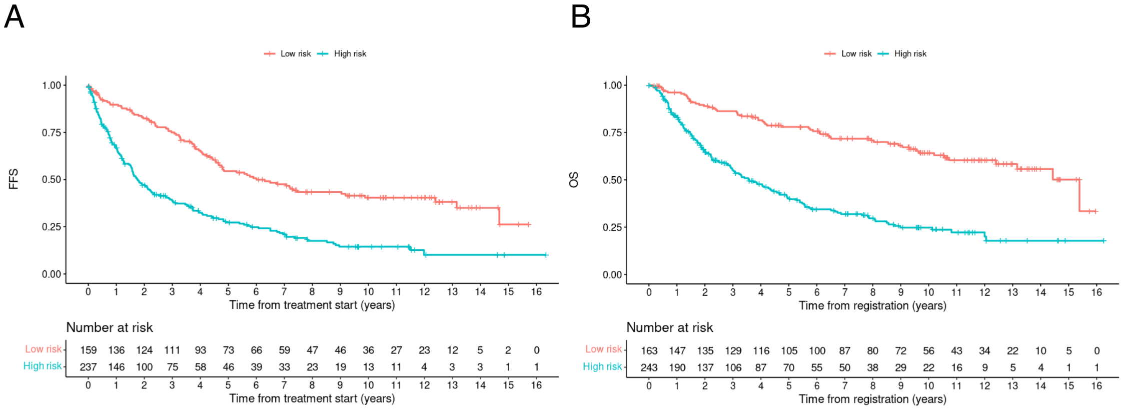

Supplemental Figure 5: Prognostic impact of high, high intermediate MIPI-c or high p53 expression on clinical outcome. Kaplan-Meier estimates of FFS (A) and OS (B) of patients

with high, high intermediate MIPI-c or p53 expression >50% (high-risk disease) compared to low or low intermediate MIPI-c and p53 expression ≤50% (low-risk disease).
